# Novel angiotensin I-converting enzyme inhibitory peptides derived from an edible mushroom, *Pleurotus cystidiosus* O.K. Miller identified by LC-MS/MS

**DOI:** 10.1186/1472-6882-13-313

**Published:** 2013-11-11

**Authors:** Ching Ching Lau, Noorlidah Abdullah, Adawiyah Suriza Shuib

**Affiliations:** 1 Mushroom Research Centre, Institute of Biological Sciences, Faculty of Science, University of Malaya, Kuala Lumpur 50603, Malaysia; 2 Medical Biotechnology Laboratory, University of Malaya Centre for Proteomics Research (UMCPR), Faculty of Medicine, University of Malaya, Kuala Lumpur 50603, Malaysia

**Keywords:** Abalone mushroom, Antihypertensive peptide, Competitive ACE inhibitor

## Abstract

**Background:**

Angiotensin I-converting enzyme (ACE) inhibitors have been reported to reduce mortality in patients with hypertension. Compared to chemosynthetic drugs, ACE inhibitors derived from natural sources such as food proteins are believed to be safer for consumption and to have fewer adverse effects. Some edible mushrooms have been reported to significantly reduce blood pressure after oral administration. In addition, mushrooms are known to be rich in protein content. This makes them a potential source of ACE inhibitory peptides. Hence, the objective of the current study was to isolate and characterise ACE inhibitory peptides from an edible mushroom, *Pleurotus cystidiosus*.

**Methods:**

ACE inhibitory proteins were isolated from *P. cystidiosus* based on the bioassay guided purification steps, i.e. ammonium sulphate precipitation, reverse phase high performance liquid chromatography and size exclusion chromatography. Active fraction was then analysed by LC-MS/MS and potential ACE inhibitory peptides identified were chemically synthesized. Effect of *in vitro* gastrointestinal digestions on the ACE inhibitory activity of the peptides and their inhibition patterns were evaluated.

**Results:**

Two potential ACE inhibitory peptides, AHEPVK and GPSMR were identified from *P. cystidiosus* with molecular masses of 679.53 and 546.36 Da, respectively. Both peptides exhibited potentially high ACE inhibitory activity with IC_50_ values of 62.8 and 277.5 μM, respectively. SEC chromatograms and BIOPEP analysis of these peptides revealed that the peptide sequence of the hexapeptide, AHEPVK, was stable throughout gastrointestinal digestion. The pentapeptide, GPSMR, was hydrolysed after digestion and it was predicted to release a dipeptide ACE inhibitor, GP, from its precursor. The Lineweaver-Burk plot of AHEPVK showed that this potent and stable ACE inhibitor has a competitive inhibitory effect against ACE.

**Conclusion:**

The present study indicated that the peptides from *P. cystidiosus* could be potential ACE inhibitors. Although these peptides had lower ACE inhibitory activity compared to commercial antihypertensive drugs, they are derived from mushroom which could be easily obtained and should have no side effects. Further *in vivo* studies can be carried out to reveal the clear mechanism of ACE inhibition by these peptides.

## Background

Angiotensin I-converting enzyme (ACE) inhibitors have been reported to reduce mortality in patients with hypertension [1]. These drugs act as vasodilators by reducing the levels of angiotensin II in the renin-angiotensin system or by inhibiting the degradation of bradykinin in the kallikrein-kinin system [2]. They have been prescribed as first-line treatment for hypertension in patients with type 1 diabetes, proteinuria or left ventricular systolic dysfunction (LVSD) [3]. Captopril was the first orally active ACE inhibitor to be synthesised [4]. Compared to chemosynthetic drugs, ACE inhibitory peptides derived from natural sources such as food proteins are believed to be safer for consumption and to have fewer adverse effects. Many ACE inhibitory peptides have been isolated from food proteins such as salmon, tuna, rice, buckwheat, soybean and whey [5-10]. Some of these ACE inhibitory peptides have exhibited stability against gastrointestinal digestion and produce a blood pressure-lowering effect when tested *in vivo*[6,8].

Mushrooms have received increasing attention in recent years because of their health-stimulating properties and medicinal effects. Some edible mushrooms have been reported to significantly reduce blood pressure after oral administration. Examples are *Pleurotus cornucopiae*, *Lyophyllum decastes*, *P. nebrodensis*, *Grifola frondosa*, *P. sajor-caju* and *Lentinula edodes*[11-16]. The protein content in mushrooms is ranked below most animal meats but above most other foods, such as milk, vegetables and fruits [17]. Thus, this makes them a good starting material for the identification of peptides with biological activities including ACE inhibition activity. ACE inhibitory peptides have been successfully purified from edible mushrooms, such as *G. frondosa*, *P. cornucopiae*, *Pholiota adiposa* and *Tricholoma giganteum*[18-21]. Among the most common edible mushrooms available in Malaysia, *P. cystidiosus* has exhibited the most potent ACE inhibitory activity. Proteomic analysis of *P. cystidiosus* has shown that it contains potential ACE inhibitory peptides [22]. Therefore, the objective of the current study was to isolate and characterise ACE inhibitory peptides from *P. cystidiosus*.

## Methods

### Materials

Sporocarps (or fruiting bodies) of *P. cystidiosus* were obtained from Gano Farm Sdn. Bhd. and authenticated by morphology and molecular methods by experts in the Mushroom Research Centre, University of Malaya, Malaysia. Herbarium voucher specimen (KLU-M 1234) was deposited in the Kuala Lumpur Herbarium, University of Malaya. Culture for this species was deposited at Mushroom Research Centre culture collection, University of Malaya and was assigned a culture code (KUM 61204). All solvents and chemicals used in this study were of analytical and HPLC grade. Acetonitrile and trifluoroacetic acid (TFA) were obtained from Merck (Darmstadt, Germany). ACE from rabbit lung, hippuryl-L-histidyl-L-leucine (HHL) and gastrointestinal proteases (pepsin, trypsin and α-chymotrypsin) were purchased from Sigma-Aldrich (St. Louis, MO, USA).

### Purification of potential ACE inhibitory peptides by size exclusion chromatography (SEC)

Protein extraction from *P. cystidiosus* was done based on a previous study [22]. Briefly, 1000 g of fresh fruiting bodies of *P. cystidiosus* were cleaned, sliced and blended with distilled water at a ratio of 1:2 (w/v). The mixture was filtered and centrifuged to remove unwanted debris. Proteins were precipitated out from the water extract using ammonium sulphate at 10-100% salt saturation. Precipitated proteins showing the highest ACE inhibitory activity were then fractionated by reverse phase high performance liquid chromatography (RPHPLC). Based on the results reported by Lau et al., [22], the active RPHPLC fraction was E5PcF3. Thus, it was further purified in the current study by SEC using a Biosep SEC-S2000 column (300 × 7.8 mm, Phenomenex, Torrance, CA, USA). Analysis was performed by injecting 20 μl of E5PcF3 on an HPLC system equipped with an SCL-10AVP system controller, LC-10ATVP solvent delivery unit, SPD-M10AVP UV–vis diode array detector and DGU-12A degasser (Shimadzu, Kyoto, Japan). The mobile phase consisted of 45% acetonitrile containing 0.1% TFA. The flow rate was 1.0 ml/min and the effluent was monitored at 214 nm. E5PcF3 was fractionated according to the peaks obtained. After repeated injections, the fractions collected were freeze-dried and the ACE inhibitory activity of the SEC fractions was determined at a concentration of 1 μg/ml protein. The SEC fraction with the highest ACE inhibitory activity was analysed by liquid chromatography mass spectrometry for sequence identification.

### Estimation of the protein content in the SEC protein fraction

The protein content of the SEC fractions was estimated using the Pierce® Bicinchoninic Acid (BCA) Protein Assay Kit (Thermo Scientific, Rockford, IL, USA) according to the protocol provided by the manufacturer. The absorbance values were measured using a Sunrise™ ELISA microplate reader (Tecan, Grödig, Austria) at 562 nm. The protein content was determined by comparing the absorbance value of the samples with a standard curve of bovine serum albumin.

### Assay of ACE inhibitory activity

In the current study, ACE inhibitory activity was determined using an ACE inhibitory assay kit (ACE kit-WST, Dojindo Laboratories, Kumamoto, Japan). The assay was carried out according to the protocol provided by the manufacturer. Absorbances of the reactions were measured using a Sunrise® ELISA microplate reader (Tecan, Grödig, Austria) at 450 nm. The ACE inhibitory activity of the samples was calculated using the formula given in the protocol. The concentration of the ACE inhibitor required to inhibit 50% of ACE activity under the above assay conditions was defined as the IC_50_.

### Liquid chromatography-mass spectrometry (LC-MS/MS)

Identification of the peptide sequences present in SEC fraction 1 was carried out by LC-MS/MS at Proteomics International Pty Ltd, WA, Australia. Briefly, the SEC fraction was digested with trypsin and the peptides extracted were analysed by electrospray ionisation mass spectrometry using an Ultimate 3000 nano HPLC system (Dionex, Sunnyvale, CA, USA) coupled to a 4000 QTRAP mass spectrometer (Applied Biosystems, Foster City, CA, USA). Peptides were loaded onto a C18 PepMap100, 3 μm (LC Packings) column and separated with a linear gradient of water/acetonitrile/0.1% formic acid (v/v). Protein identification was carried out using Mascot sequence matching software (Matrix Science) with the Ludwig NR database.

### Peptide synthesis

The two identified potential ACE inhibitory peptides, AHEPVK and GPSMR were chemically synthesised by Peptron, Inc., Republic of Korea. The purity of the synthesised peptides was >98% measured by RPHPLC and MS analysis.

### Effect of simulated gastrointestinal digestion on the selected peptides

The stability of the synthesised peptides against gastrointestinal proteases was assessed *in vitro* by the method of Wu and Ding [23]. The peptide solution (0.1 mg/ml, 0.5 ml) was incubated with 0.5 ml of a 0.05% pepsin solution (0.1 M HCl at pH 2.0) for 2.5 hrs at 37°C. In the successive pepsin-pancreatin digestion test, the peptide solution was adjusted to pH 8.0 after pepsin digestion. Then, 0.5 ml of pancreatin solution [potassium phosphate buffer (0.1 M, pH 8.0) containing 0.025% (w/v) chymotrypsin and 0.025% (w/v) trypsin] was added to the solution. The mixture was incubated for another 2.5 hrs at 37°C. The control (without digestion) consisted of peptide solution incubated in buffer solutions (HCl and potassium phosphate buffer) and was carried out alongside the experiment. After enzymatic treatment, the pepsin solution and pepsin-pancreatin solution were boiled for 10 min to stop the digestion and then centrifuged at 10,000 rpm for 10 min. The supernatants were freeze-dried and used for the measurement of ACE inhibitory activity. The stability of the purified peptides against gastrointestinal enzymes was analysed by SEC.

### Determination of the inhibition pattern on ACE activity

The inhibition pattern of peptide AHEPVK on ACE activity was determined spectrophotometrically using HHL as substrate. Basically, 20 μl of the ACE solution (0.1 UN/ml) and 50 μl of peptide were incubated with 200 μl of various HHL concentrations (0.63, 1.25, 2.50 and 5.00 mM). The enzymatic reaction was terminated by the addition of 250 μl of 1.0 M HCl. The liberated hippuric acid was extracted with ethyl acetate and evaporated under vacuum condition. The hippuric acid residue was re-dissolved in 1.0 ml of distilled water and the absorbance was determined at 228 nm using a spectrophotometer (SmartSpec™ Plus Spectrophotometer, Bio-Rad Laboratories, Hercules, USA). The enzyme activities were measured in the presence (0.05 and 0.50 mg/ml) and absence (control) of peptide. The kinetic of ACE inhibition was determined by Lineweaver-Burk plots.

### Statistical analysis

The analysis of ACE inhibitory activity was carried out in triplicate and result was reported as mean ± standard deviation. Mean differences of ACE inhibitory activity in SEC fractions was analyzed using one-way ANOVA in Statgraphics Plus 3.0 at p < 0.05.

## Results and discussion

### Purification of potential ACE inhibitory peptides by SEC

**Figure 1:**
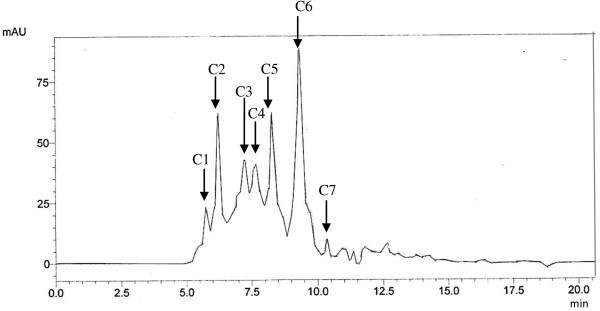
**SEC chromatogram of E5PcF3.** Following RPHPLC, active protein E5PcF3 was further separated using a Biosep SEC-S2000 column (300 × 7.8 mm). The mobile phase consisted of 45% acetonitrile containing 0.1% TFA eluted at a flow rate of 1.0 ml/min. Seven peaks eluted from SEC column labelled C1 to C7 were collected and re-evaluated for ACE inhibitory activity.

The RPHPLC fraction of E5PcF3 was further fractionated by SEC into seven fractions (C1 to C7), as shown in Figure 1. Referring to Table 1, a total of 83.4% of the proteins were recovered by SEC. The percentages of protein collected from fractions C1 to C7 were in the range of 3.6 to 24.6%. Each SEC fraction was tested for ACE inhibitory activity at a concentration of 1 μg/ml. Among the seven SEC fractions, C1 exhibited significantly higher ACE inhibitory activity, where 27.44% of ACE enzyme activity was blocked. Therefore, C1 was selected for further analysis by LC-MS/MS.

**Table 1:** Percentages of protein recovery yield and percentages of ACE inhibitory activity of the SEC fractions

**SEC fraction**	**% Recovery**	% ACE inhibitory activity^*^
**C1**	**3.6**	**27.44 ± 2.66 c**
C2	3.9	5.56 ± 2.18 a
C3	24.6	7.47 ± 0.82 a
C4	12.8	7.98 ± 4.72 a
C5	9.6	5.93 ± 2.24 a
C6	12.3	8.70 ± 2.54 ab
C7	16.6	13.60 ± 3.99 b
Total	83.4	-

^*^ACE inhibitory activity of SEC fractions was tested at 1 μg/ml protein and expressed as mean ± standard deviation (n = 3). Different letters within a column indicate significant differences (p < 0.05) in the percentage of ACE inhibitory activity analysed by one-way ANOVA. SEC fraction highlighted in bold was selected for further analysis.

### Identification of ACE inhibitory peptide by LC-MS/MS

**Figure 2:**
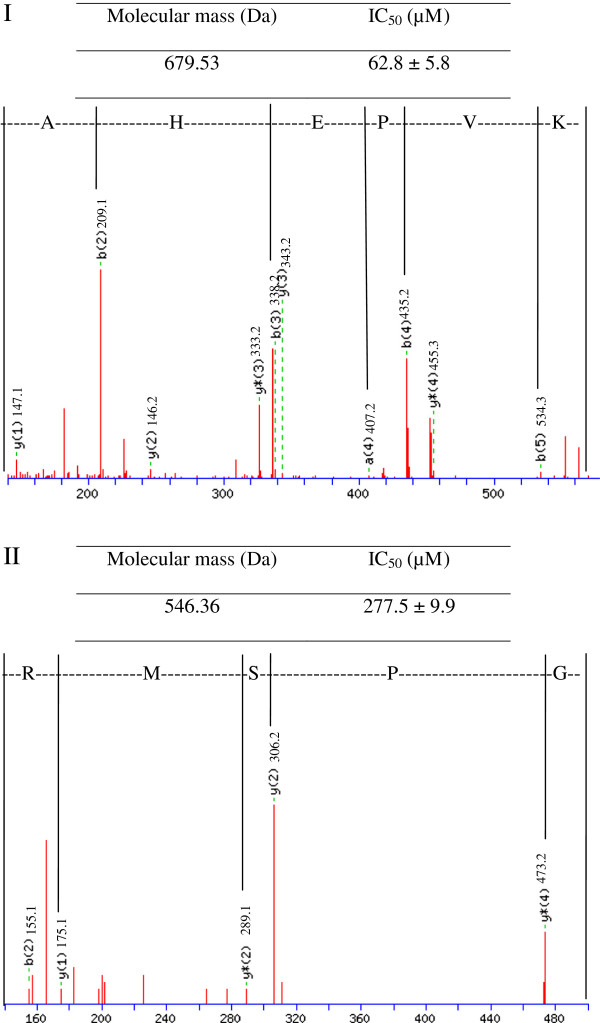
**LC-MS/MS spectra of peptides (I) AHEPVK and (II) GPSMR with the estimated molecular mass and IC**_**50**_**value of ACE inhibitory activity.**

The amino acid sequences of the peptides in C1 were determined by LC-MS/MS. Two potential ACE inhibitory peptides were identified. The LC-MS/MS spectra of these peptides are shown in Figure 2. Peptides AHEPVK and GPSMR had molecular masses of 679.53 and 546.36 Da, respectively. A low molecular weight is an added advantage for a potent ACE inhibitor because large peptide molecules are restricted from fitting into the active site of ACE [24]. Interestingly, the two peptides in the current study were found to have similar sequence compared to ACE inhibitory peptides from other food sources. For instance, similar to AHEPVK, potential ACE inhibitor from sea squirt (AHIII) has alanine and histidine at the N-terminal [25]. GPSMR has similar peptide sequence with peptide from sweet potato (GPCSR) [26].

In the current study, peptide AHEPVK exhibited potentially high ACE inhibitory activity with an IC_50_ value of 62.8 μM. This is lower than the IC_50_ value of ACE inhibitory peptides isolated from other edible mushrooms, i.e. *G. frondosa* (129.7 μM), *P. adiposa* (254 μM) and *P. cornucopiae* (277.3 μM) [18,20,21]. On the other hand, peptide GPSMR inhibited 50% of ACE activity at a concentration of 277.5 μM, which is similar to the IC_50_ values of *P. adiposa* and *P. cornucopiae*[18,20].

The peptides in the current study have lower ACE inhibitory activity than proteins from other food sources. Hexapeptide from milk (PYVRYL) and pentapeptide from bonita fish (LKPNM) have exhibited IC_50_ value of 2.4 μM [27,28]. Peptide LRIPVA from spinach inhibited 50% of the ACE at a concentration as low as 0.38 μM [29]. However, there were also studies reported on similar IC_50_ value with the current study. Peptides GTEKC and GPCSR from sweet potato exhibited IC_50_ values of 61.67 and 275.8 μM, respectively [26,30].

There are several ACE inhibitory peptides from food sources that have been sold in Japan and Canada. Examples are Vasotensin®, PeptACE™ and Valtyron® [31]. These products were claimed to be suitable for treating mild hypertension and free of side effects [32,33]. This may suggest that ACE inhibitors from natural sources can be a good alternative to synthetic drugs which are known to cause side effects such as cough, skin rashes, taste disturbance and angioedema [34]. Even though peptides from edible mushrooms have lower ACE inhibitory activity compared to peptides from other food sources, *in vivo* studies of peptides from mushrooms, *P. adiposa* and *P. cornucopiae* have shown similar antihypertensive effect with the commercial drug, captopril [18,20]. Additionally, mushrooms have an added advantage of low probability to cause food allergy. Therefore, the peptides tested in the current study, particularly AHEPVK could be applied as ingredient in functional foods, dietary supplements or pharmaceuticals as an antihypertensive agent.

### Effect of simulated gastrointestinal digestion on the selected peptides

Proteins or peptides delivered by the oral route have to be able to maintain their biological activity throughout the digestion process in the gastrointestinal tract before they reach their target site inside the body. The most important sites for the digestion of proteins and peptides are the stomach and small intestine. They contain gastrointestinal enzymes such as pepsin (stomach), trypsin and chymotrypsin (small intestine) [35]. Preliminary experiments using gastrointestinal enzyme incubation *in vitro* provided an easy method to evaluate the fate of these peptides after oral administration.

**Figure 3:**
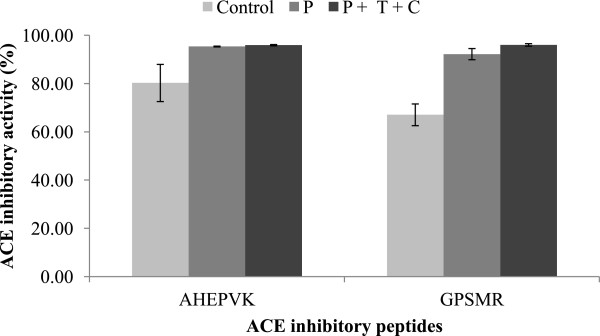
**Effect of simulated gastrointestinal digestion on the ACE inhibitory activity of peptides AHEPVK and GPSMR.** Control: The peptide solutions (0.1 mg/ml) were incubated in buffer solutions (HCl and potassium phosphate buffer). P: The peptide solutions were incubated with 0.05% pepsin solution for 2.5 hrs at 37°C. P + T + C: The peptide solutions were successively digested with pepsin for 2.5 hrs. They were further incubated in pancreatin solution for another 2.5 hrs at 37°C. The ACE inhibitory activity are expressed as mean ± standard deviation (n = 3).

Referring to Figure 3, both peptides had exhibited high ACE inhibitory activity after gastrointestinal digestion. Without gastrointestinal digestion, AHEPVK inhibited 80.27% of ACE activity. Its activity was enhanced to 95.38% after digestion by pepsin and maintained at 95.94% after pepsin-pancreatin digestion. Compared to AHEPVK, GPSMR exhibited greater enhancement after digestion. The ACE inhibitory activity increased from 67.08% to 92.22% after digestion by pepsin. Pepsin-pancreatin digestion further enhanced the ACE inhibitory activity to 96.05%. Previous studies have reported on peptides which were resistant to further gastrointestinal digestion and maintain their biological activity after digestion [36]. However, some peptides could undergo further hydrolysis by gastrointestinal enzymes to release true inhibitors [37].

**Figure 4:**
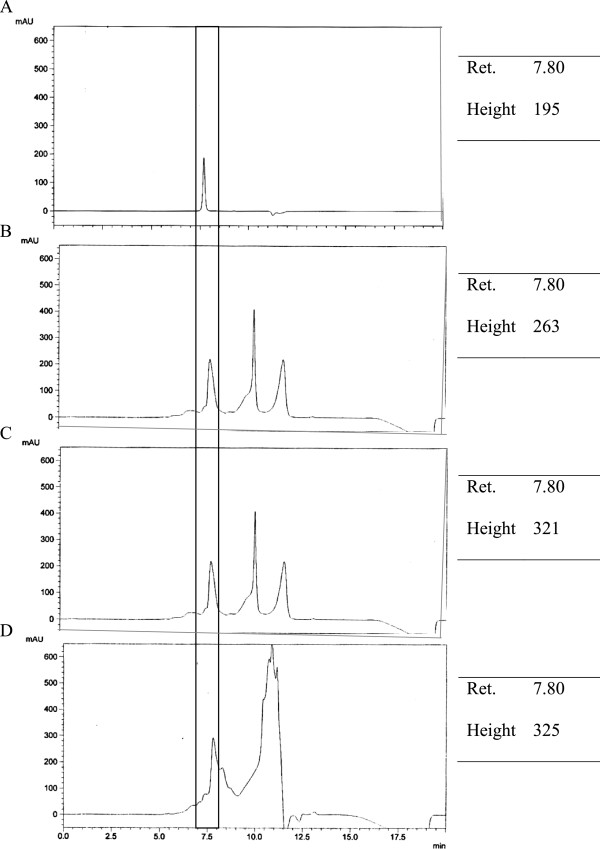
**Stability of peptide AHEPVK against gastrointestinal enzymes observed by SEC chromatograms.** Separation was performed on a Biosep SEC-S2000 column (300 × 7.8 mm). Mobile phase consisted of 45% acetonitrile containing 0.1% TFA eluted at a flow rate of 1.0 ml/min. Peptide was eluted as peak at retention time illustrated in the box. **A**: Pure peptide; **B**: The peptide solution (0.1 mg/ml) was incubated in buffer solutions (control); **C**: The peptide solution was incubated with 0.05% pepsin solution for 2.5 hrs at 37°C; **D**: The peptide solution was successively digested with pepsin for 2.5 hrs. They were further incubated in pancreatin solution for another 2.5 hrs at 37°C.

**Figure 5:**
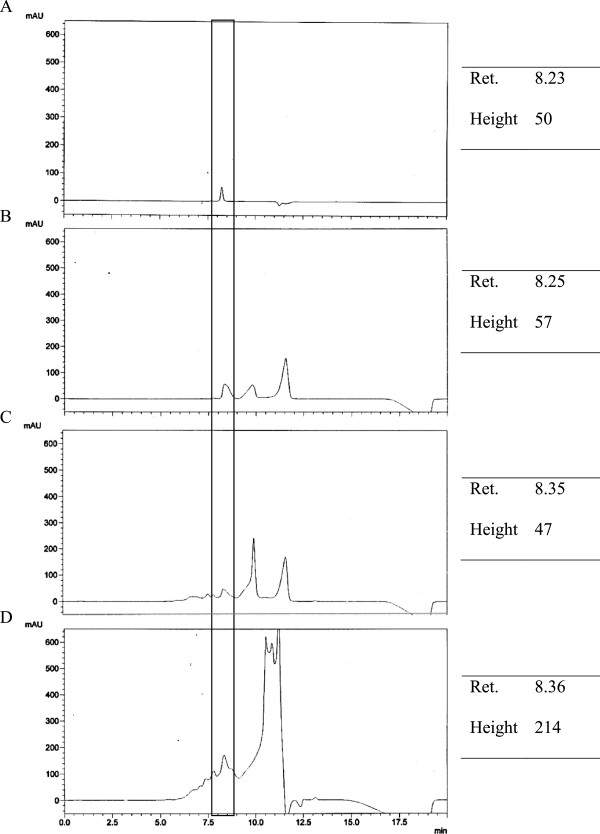
**Stability of peptide GPSMR against gastrointestinal enzymes observed by SEC chromatograms.** Separation was performed on a Biosep SEC-S2000 column (300 × 7.8 mm). Mobile phase consisted of 45% acetonitrile containing 0.1% TFA at a flow rate of 1.0 ml/min. Peptide was eluted as peak at retention time illustrated in the box. **A**: Pure peptide; **B**: The peptide solution (0.1 mg/ml) was incubated in buffer solutions (control); **C**: The peptide solution was incubated with 0.05% pepsin solution for 2.5 hrs at 37°C; **D**: The peptide solution was successively digested with pepsin for 2.5 hrs. They were further incubated in pancreatin solution for another 2.5 hrs at 37°C.

In order to verify the stability of these peptides, the changes without and following gastrointestinal digestion were analysed by SEC. The chromatograms are illustrated in Figures 4 and 5. Peaks for buffer (HCl and potassium phosphate buffer) were eluted at approximately 9 and 11 min. This may explained the detection of two extra peaks in the chromatograms. The BIOPEP database (http://www.uwm.edu.pl/biochemia/index.php/en/biopep) is an online program that can serve as a tool to predict possible proteolysis products by gastrointestinal enzymes and define the possible biological activity of the proteolysis fragments [38]. Therefore, the predicted proteolysis activity analysed by the BIOPEP database was compared with the SEC chromatograms of AHEPVK and GPSMR in the current study.

According to BIOPEP, AHEPVK was not hydrolysed by the three proteolytic enzymes. It was predicted to remain stable throughout the digestion process. Referring to Figure 4, the peptide AHEPVK, which was eluted at 7.80 min, showed high intensity in the SEC chromatograms of the control and after digestion. This confirmed the stability of AHEPVK against digestive enzymes. Additionally, Wang et al. [39] have reported that the preferential parameters for hexapeptides with potent ACE inhibitory activity are stereo and hydrophobic properties. Jimsheena and Gouda had shown the important role of stereo-specificity of amino acid residue in ACE inhibitory activity. Based on their study, tripeptide IKP that contained L-lysine exhibited potent ACE inhibitory activity. However, replacement of the L-lysine with D-lysine caused the peptide to lose its ACE inhibitory property [40]. Hydrophobicity of amino acids has been indicated to have the greatest influence on ACE inhibitory activity. According to Pripp and co workers, hydrophobicity of C-terminal enhanced the ACE inhibitory activity of potential peptides up to six amino acids in length [41]. In the current study, the stereoisomer effect of AHEPVK on ACE inhibition was not definitive due to the unknown stereo structure of the synthesized peptide. However, based on the peptide sequence, hydrophobicity may have contributions in the high ACE inhibitory activity of AHEPVK both before and after digestion.

Referring to Figure 5, the peptide peak of GPSMR at a retention time of 8.23 min was shifted and became broader after gastrointestinal digestion. Theoretically, smaller peptides would be eluted from the SEC column at a later time [42]. This may suggest that the peptide GPSMR had been hydrolysed into smaller fragments that were eluted together with gastrointestinal enzymes, resulting in a broad peak at 8.36 min. This is in line with the results obtained by BIOPEP analysis. According to the database, GPSMR was predicted to release fragments of GP, SM and R from its precursor after gastrointestinal digestion. Interestingly, dipeptide GP has been previously reported to exhibit ACE inhibitory activity with an IC_50_ value of 252.63 μM [43]. Therefore, the enhanced ACE inhibitory activity of GPSMR after gastrointestinal digestion was most probably due to the release of GP.

### Inhibition pattern of ACE inhibitors

**Figure 6:**
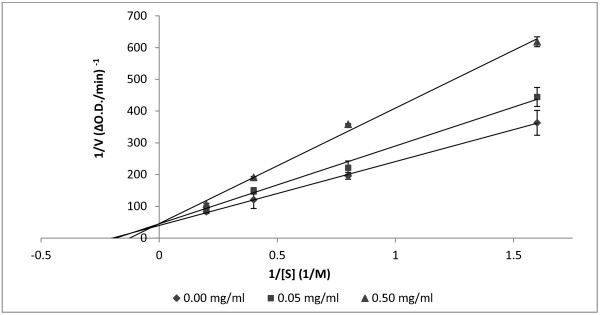
**Kinetics of the synthetic peptide AHEPVK.** ACE inhibitory activity was determined in the absence and presence of different concentrations of the peptides (0.00, 0.05 and 0.50 mg/ml). Lineweaver-Burk plot was constructed using values of 1/v against 1/ [S]. Values are expressed as mean ± standard deviation (n = 3).

Peptide AHEPVK exhibited the most potent ACE inhibitory activity (IC_50_ 62.8 μM) and it shows stability against gastrointestinal digestion. Therefore, it was selected to determine its inhibition pattern against the ACE enzyme. According to the Lineweaver-Burk plot in Figure 6, peptide AHEPVK showed a competitive inhibition pattern against the ACE. This suggests that the peptide might bind to the active site of ACE to block it from binding to the substrate. Moreover, ACE has been reported to show preference for competitive inhibitors that contain a hydrophobic amino acid at the third position from the C-terminal [44,45]. This is in accordance with the amino acid sequence of AHEPVK which might explain the competitive inhibition pattern exhibited by this peptide.

The competitive inhibition pattern exhibited by AHEPVK is similar to ACE inhibitory peptides purified from the edible mushrooms *G. frondosa*, *P. cornucopiae*, *P. adiposa* and *T. giganteum*[18-21]. In addition, a commercial ACE inhibitor and antihypertensive drug, captopril, also inhibits ACE in a competitive manner [4].

## Conclusion

In the current study, peptides isolated from *P. cystidiosus* were shown to be potential ACE inhibitors. Peptide AHEPVK exhibited a high IC_50_ value (62.8 μM) and its peptide sequence remained stable following gastrointestinal digestion. It exhibited a competitive inhibition pattern against ACE. Peptide GPSMR was predicted to release a dipeptide ACE inhibitor, GP, from its precursor after gastrointestinal digestion. Although these peptides had lower ACE inhibitory activity compared to commercial antihypertensive drugs, they are derived from food sources and should have no side effects.

## Abbreviations

ACE: Angiotensin I-converting enzyme; RPHPLC: Reverse phase high performance liquid chromatography; SEC: Size exclusion chromatography; LC-MS/MS: Liquid chromatography mass spectrometry.

## Competing interests

The authors declare that they have no competing interests.

## Authors’ contributions

CCL carried out all the experimentation, analysis of data and drafting of the manuscript. NA involved in monitoring and coordinating the work on mushroom biology and antihypertensive activity. ASS involved in coordinating the work on isolation and purification of peptides; and proteomic analysis. All authors read and approved the final manuscript.

## Pre-publication history

The pre-publication history for this paper can be accessed here:

http://www.biomedcentral.com/1472-6882/13/313/prepub
